# Guidance on review type selection for health technology assessments: key factors and considerations for deciding when to conduct a *de novo* systematic review, an update of a systematic review, or an overview of systematic reviews

**DOI:** 10.1186/s13643-022-02071-7

**Published:** 2022-09-27

**Authors:** Joanne S. M. Kim, Michelle Pollock, David Kaunelis, Laura Weeks

**Affiliations:** 1 Canadian Agency for Drugs and Technologies in Health, Ottawa, ON Canada; 2 Institute of Health Economics, Edmonton, AB Canada

**Keywords:** Systematic review, Review update, Overview of reviews, Evidence synthesis, Health technology assessment, Decision tool, Review type selection

## Abstract

**Background:**

A systematic review (SR) helps us make sense of a body of research while minimizing bias and is routinely conducted to evaluate intervention effects in a health technology assessment (HTA). In addition to the traditional de novo SR, which combines the results of multiple primary studies, there are alternative review types that use systematic methods and leverage existing SRs, namely updates of SRs and overviews of SRs. This paper shares guidance that can be used to select the most appropriate review type to conduct when evaluating intervention effects in an HTA, with a goal to leverage existing SRs and reduce research waste where possible.

**Process:**

We identified key factors and considerations that can inform the process of deciding to conduct one review type over the others to answer a research question and organized them into guidance comprising a summary and a corresponding flowchart. This work consisted of three steps. First, a guidance document was drafted by methodologists from two Canadian HTA agencies based on their experience. Next, the draft guidance was supplemented with a literature review. Lastly, broader feedback from HTA researchers across Canada was sought and incorporated into the final guidance.

**Insights:**

Nine key factors and six considerations were identified to help reviewers select the most appropriate review type to conduct. These fell into one of two categories: the evidentiary needs of the planned review (i.e., to understand the scope, objective, and analytic approach required for the review) and the state of the existing literature (i.e., to know the available literature in terms of its relevance, quality, comprehensiveness, currency, and findings). The accompanying flowchart, which can be used as a decision tool, demonstrates the interdependency between many of the key factors and considerations and aims to balance the potential benefits and challenges of leveraging existing SRs instead of primary study reports.

**Conclusions:**

Selecting the most appropriate review type to conduct when evaluating intervention effects in an HTA requires a myriad of factors to be considered. We hope this guidance adds clarity to the many competing considerations when deciding which review type to conduct and facilitates that decision-making process.

## Background

A systematic review (SR) helps us make sense of a body of research, while minimizing bias, by identifying, analysing, appraising, synthesizing, and interpreting research findings using a standardized, pre-defined method and an a priori protocol [1]. In the context of health care, the SR methodology provides an essential tool for summarizing the available published knowledge around a specific research question to support organizations and professionals who make recommendations and decisions about wide-ranging issues [2, 3]. In health technology assessments (HTAs), which use explicit methods to determine the value of a health technology at different points in its lifecycle [4], SRs are routinely conducted to answer research questions around the effectiveness and safety of drugs, medical devices, and clinical interventions and provide clinical evidence to support their appropriate use. This paper focuses on such use of SRs of quantitative research in HTAs, that is, in the evaluation of interventions to prevent, diagnose, or treat diseases or conditions.

The traditional de novo SR combines the results of multiple primary studies to answer a specific research question [5, 6]. There are, however, two alternative review types that use systematic methods and leverage existing SRs, namely an update of an SR and an overview of SRs. An update of an SR incorporates new evidence (by extending the source- or time-related domains of its search, including additional databases or longer periods), new methods, or new analyses into a previously completed SR [6–8]. An overview of SRs—also known as a review of reviews, an umbrella review, a meta-review, or other variations of these terms—compiles data from multiple SRs to provide a single summary of relevant evidence [5, 9]. These three review types—de novo SRs, updates of existing SRs, and overviews of existing SRs—are most often used to evaluate intervention effects and form the basis of clinical evidence in HTAs. As such, other review types (e.g., scoping reviews, integrative reviews, and realist reviews) [10, 11] were out of scope for this paper. Rapid reviews [12] and living reviews [13] were also out of scope for this paper, as these approaches can be used with any review type (e.g., an overview may be conducted rapidly with a single reviewer and updated regularly and frequently as a living document).

**Table:** 

**Definitions**	
*de novo* SR: uses systematic and explicit methods* to combine the results of multiple primary studies to answer a specific research question [5, 6].	
Update of an SR: uses systematic and explicit methods* to incorporate new evidence, new methods, or new analyses into a previously completed SR﻿ [6–8].	
Overview of SRs: uses systematic and explicit methods* to compile data from multiple SRs to provide a single summary of relevant evidence [5, 9].	
*Systematic and explicit methods usually involve: publishing a priori protocols that are detailed enough to be reproducible by others; searching multiple databases and grey literature to identify as much of the available relevant data as possible; and engaging two reviewers for each step during the review process to minimize bias.	

Given the exponential growth in the number of published SRs [14, 15] and high-resource requirements for completing new SRs [15, 16], conducting updates or overviews of existing SRs, instead of de novo SRs, may be considered by reviewers under the right circumstances. Using existing SRs in updates or overviews can help minimize duplication of effort and redundancy in research and can contribute to a more efficient use of resources and a further synthesis of the extant literature [17]. However, there are unique challenges associated with conducting updates or overviews of existing SRs, arising from the need to rely on the work of the SR authors, which can be resource-intensive to address [15]. In other words, there are trade-offs. Therefore, potential benefits and challenges of leveraging existing SRs instead of primary study reports must both be considered. While there is some guidance on how to select a review type to conduct [2, 15, 18–20], we are not aware of guidance that incorporates the myriad of factors that we have come to consider when making that decision in our work.

The objective of this paper is to share our guidance that is based on our work. It can be used when selecting the most appropriate review type that uses systematic methods to answer a research question about intervention effects in an HTA, while ensuring an appropriate balance between evidentiary needs and methodological rigour. The specific goals were as follows: (1) to identify factors that should be considered when deciding to conduct a de novo SR, an update of an existing SR, or an overview of existing SRs, and (2) to develop an algorithm to help with that decision-making process. The intent of this paper is not to provide a definitive answer on which review type to conduct in a specific scenario or to offer guidance on how to conduct a review using a specific review type. Rather, this paper identifies key factors that should be considered and organizes them into a summary and a flowchart that can be used as a decision tool to identify the right circumstances in which one review type over the others may be favoured or ruled out.

## Process

This work consisted of three steps. First, a guidance document was drafted by methodologists from two Canadian HTA agencies based on their experience. Next, the draft guidance was supplemented with a literature review. Lastly, broader feedback from HTA researchers across Canada was sought and incorporated into the final guidance.

### Step 1. Draft guidance

The Canadian Agency for Drugs and Technologies in Health (CADTH) and the Institute of Health Economics (IHE) have conducted both updates and overviews of existing SRs, instead of de novo SRs, to answer clinical research questions in several HTAs (e.g., see references [21–25]). Methodologists at CADTH (JK, LW) and IHE (MP), who had been involved in deciding which review types to conduct for the abovementioned projects, reflected on the key factors and considerations that informed, or could have informed, those decisions and organized them into a guidance document that comprised a summary and a corresponding flowchart outlining the decision-making process. The guidance was originally drafted by the methodologists at CADTH and then shared with the methodologist at IHE for review and input. This collaboration across the two HTA organizations helped ensure that the guidance was both methodologically robust and applicable across different decision-making contexts. Thus, the draft guidance reflected the experiences and expertise of two Canadian HTA agencies (i.e., CADTH and IHE).

### Step 2. Literature review

A literature review was conducted to identify any existing related guidance from academia, SR organizations, and other HTA agencies.

#### Search strategy

A targeted literature search was performed by an information specialist at CADTH. Published literature was identified by searching the MEDLINE database via Ovid. The search strategy was comprised of both controlled vocabulary, such as the National Library of Medicine’s MeSH (Medical Subject Headings), and keywords. Retrieval was not limited by publication date but was limited to the English language. The search was completed on March 5, 2019. Regular alerts updated the search until manuscript submission. The complete search strategy is presented in Appendix 1.

Grey literature (literature that is not commercially published) was identified by searching sources listed in relevant sections of the *Grey Matters* checklist (https://cadth.ca/grey-matters-practical-tool-searching-health-related-grey-literature) [26], primarily the websites of Canadian and major international HTA agencies. Google was used to search for additional web-based materials. These searches were supplemented by reviewing bibliographies of key papers. More information on the grey literature search strategy is presented in Appendix 2.

#### Screening and selection

Literature identified through the database and grey literature searches were screened by a single reviewer (JK) at CADTH. Guidance for deciding when to conduct a de novo SR, an update of an existing SR, or an overview of existing SRs to evaluate intervention effects was in scope. Further, the following eligibility criteria were applied:

- Documents of the following nature were in scope and included:
  - ○ Provide explicit guidance for selecting one review type over another
  - ○ Describe when it is or is not appropriate to use a specific review type
  - ○ Help identify, define, or set thresholds for factors and considerations important for deciding to conduct a specific review type (e.g., how to judge relevance, quality, or currency of an existing SR)
- Documents of the following nature were not in scope and excluded:
  - ○ Papers on review types that are not intended to answer research questions about intervention effects (e.g., scoping reviews, integrative reviews, or realist reviews)
  - ○ Papers on methodological approaches (e.g., rapid reviews or living reviews) that can be used with any review type
  - ○ Primary studies, SRs, or clinical practice guidelines on specific topics and their protocols
  - ○ Guidance for conducting a review using a specific review type
  - ○ Guidance for conducting SRs in specific fields (e.g., anatomy or nursing)
  - ○ Guidance for integrating quantitative and qualitative evidence (e.g., mixed methods or for knowledge translation)
  - ○ Anecdotal, editorial, or opinion-based literature

#### Data extraction and analysis

Text that provided explicit guidance for selecting one review type over another, described when it is or is not appropriate to use a specific review type, or helped identify, define, or set thresholds for factors and considerations important for deciding to conduct a specific review type was deemed relevant. Excerpts of relevant text from the included documents were extracted and organized by a single reviewer (JK) at CADTH according to the factors and considerations identified in Step 1. All excerpts of relevant text are presented in Appendix 3.

#### Synthesis

The excerpts of relevant text in Appendix 3 were used to confirm, support, complement, or refine the draft guidance from Step 1. Details and citations from the literature review were incorporated into the guidance to help ensure that our guidance agreed with existing guidance from the literature. The updated guidance was then shared a second time with the methodologist at IHE (MP) for review and input.

### Step 3. Broader discussion and feedback

The updated guidance from Step 2, which incorporated CADTH’s and IHE’s experiences as well as existing guidance from the literature, was shared more broadly with HTA researchers across Canada who have experience with deciding which review types to conduct and with conducting clinical reviews in HTAs. Input was collected from an additional nine researchers at CADTH and IHE as well as from 13 researchers at the Institut national d'excellence en santé et en services sociaux (INESSS) and the National Advisory Committee on Immunization (NACI). This input was collected through written feedback and/or small group discussions and further incorporated into the updated guidance from Step 2. Thus, the final guidance reflected the experiences and expertise of several Canadian HTA agencies (i.e., CADTH, IHE, INESSS, and NACI). All authors reviewed and approved the final version of the guidance, which appears in this paper.

## Insights

This section has two parts. The first part identifies key factors and considerations that can inform the decision to select the most appropriate review type to answer a research question about intervention effects in an HTA. The second part organizes those key factors and considerations into a summary and a flowchart that can guide the decision-making process.

### Part 1. Key factors and considerations

In total, nine key factors and six considerations that are important for selecting a specific review type to conduct for evaluating intervention effects were identified through reflections on experiences with clinical reviews in past HTA projects at CADTH and IHE. The literature review found these factors and considerations to be in good agreement with existing guidance from academia, SR organizations, and other HTA agencies. In total, 19 papers were identified as relevant through the literature review. They were from Joanna Briggs Institute [27], the Cochrane Collaboration [6, 9], the Agency for Healthcare Research and Quality [15, 18, 19, 28], the Belgian Health Care Knowledge Centre [29], the World Health Organization [30], eight different HTA agencies [31], the Centre for Reviews and Dissemination at the University of York [3], and other research groups [2, 8, 10, 17, 32–35] (see Appendix 3). The feedback received from other HTA researchers across Canada also found these factors and considerations to be representative of the experiences of different organizations and applicable across different decision-making contexts.

The nine key factors and six considerations each fell into one of two categories: the evidentiary needs of the planned review and the state of the existing literature. The key factors and considerations are presented in Table 1 and described below, with supporting citations. Of note, not all three review types are discussed or discussed to the same extent under each factor, as not all factors and considerations are relevant or deterministic in favouring or ruling out each review type. Also, many of the key factors and considerations are interdependent, as the evidentiary needs of a planned review must be balanced with the state of the existing literature. Therefore, many of the factors and considerations are not sufficient on their own to help select one review type over another, which motivated us to organize them into a summary and a corresponding flowchart in the following section. Further, we kept in mind the unique challenges that overviews present, stemming from having to rely on SRs, instead of primary studies, as the unit of analysis, and therefore possibly requiring more resources, compared to SRs [15, 19], and tried to balance them with the potential benefits obtained (e.g., summarizing the existing literature at higher levels or avoiding or minimizing duplication or redundancy in research).

**Table 1:** Key factors and considerations for selecting to conduct one review type over another

Key factor	Consideration
Evidentiary needs of the planned review	
1. Review scope: Is the scope of the research question broad?	I. If the planned review has narrowly defined PICO elements, a de novo SR or an update of an SR may be the most appropriate review type. If the planned review has a broader scope that expands upon one of the PICO elements, an overview of SRs may be the most appropriate review type.
2. Review objective and analytic approach: Is quantitative combination of findings needed to provide a summary measure or to rank interventions?	II. If the planned review requires quantitative combination of findings through MAs, ITC, or NMAs to provide a summary measure or to rank interventions, a de novo SR or an update of an SR should be considered instead of an overview of SRs. In an overview, a narrative, not quantitative, synthesis should be conducted.
State of the existing literature	
3. Relevance: Are there one or more relevant SRs available?	III. The quantity of relevant SRs available may rule out certain review types for the planned review. If there is no relevant SR, no overview or update could be conducted. If there is only one relevant SR, no overview should be conducted.
4. Methodological quality: Are the relevant SRs of sufficiently high quality in methodology?	IV. Ideally, only SRs of high quality in both methodology and reporting should be used for updates and, in many cases, for overviews. In the absence of high-quality SRs, replication in the form of a de novo SR may appropriate.
5. Reporting quality: Are the relevant SRs of sufficiently high quality in reporting?	
6. Comprehensiveness: Are the relevant SRs comprehensive?	V. If relevant SRs are not comprehensive or outdated and need to be supplemented with additional primary studies that are available, an overview of SRs may not be best. Instead, an update of an SR—if there is a high-quality SR available—or a de novo SR—if there is no high-quality SR available—is recommended for consistency in the analytic approach at the study level.
7. Currency: Are the relevant SRs up to date?	
8. New evidence: Are there additional relevant primary studies missing from the relevant SRs?	
9. Discordance in results: Are the findings of the relevant SRs discordant for unknown reasons?	VI. If the results of two or more relevant SRs with matching PICO elements are discordant, and reasons for discordance cannot be reliably determined, a de novo SR may be needed.

*ITC* Indirect treatment comparison, *MA* Meta-analysis, *NMA* Network meta-analysis, *PICO* Population, intervention, comparator, and outcome, *SR* Systematic review

Reviewers who plan to use these key factors and considerations to help select the most appropriate review type for their HTA will need an understanding of both the evidentiary needs of the planned review and the state of the existing literature. Specifically, there must be a clear research question with a well-defined scope. Further, a scoping exercise should be undertaken to gain a general idea of the quantity, quality, and other characteristics of relevant SRs and primary studies that are available. While it may be difficult to obtain a comprehensive understanding of the literature through the scoping exercise, and reviewers will likely work with incomplete information, the guidance provided in this paper is intended to help reviewers think through, and plan for, the issues that they are likely to encounter when deciding which review type to conduct. To that end, reviewers may find it difficult to answer the questions provided below with certainty (e.g., instead of “yes” or “no,” “likely yes,” “likely no,” or even “unclear”), especially when the existing literature is vast or complex. Nevertheless, the scoping exercise could be viewed as an “upfront” investment in the conduct of an HTA that need not be wasted (e.g., information learned through the scoping exercise can be “reused” during the review process) and may end up saving resources (e.g., by identifying an existing SR and eliminating the need for a new SR) or achieving other meaningful goals (e.g., summarizing the existing literature at higher levels or avoiding or minimizing duplication or redundancy in research).

I. Evidentiary needs of the planned review
  1. Review scope: Is the scope of the research question broad?
    A de novo SR and an update of an SR are identical or similar in scope [8], which tends to be focused and narrow [2, 34]. An overview of SRs, on the other hand, tends to have a broader scope [2, 3, 10, 32, 33, 35]. When expressed in terms of the Population, Intervention, Comparator, and Outcome (PICO) framework, a de novo SR or an update of an SR tends to have narrowly defined PICO elements, while an overview tends to have expanded upon one of the PICO elements. As described by the Cochrane Collaboration, overviews are appropriate for addressing research questions relating to:
    The same intervention used for different conditions or populations (i.e., broader P);
    Different interventions used for the same condition or population (i.e., broader I);
    Different approaches to the application of the same intervention used for the same condition or population (i.e., broader I);
    The same intervention used for the same condition or population but for different outcomes or time points (i.e., broader O); or
    Adverse effects of an intervention for one or more conditions or populations (i.e., broader O or P) [27, 32, 34].
    In other words, for an overview, there are two or more relevant SRs available that each covers one “part” of a broad research question, with all SRs combined resulting in perfect or almost perfect coverage [2, 9, 10, 27, 32–35].
  *Consideration I: If the planned review has narrowly defined PICO elements, a de novo SR or an update of an SR may be the most appropriate review type. If the planned review has a broader scope that expands upon one of the PICO elements, an overview of SRs may be the most appropriate review type.*
  2. Review objective and analytic approach: Is quantitative combination of findings needed to provide a summary measure or to rank interventions?
    A de novo SR, an update of an SR, and an overview of SRs all intend to answer a specific research question [5], using explicit, systematic methods [34]. However, the unit of searching, inclusion, data extraction, analysis, and synthesis varies from the primary study—for a de novo SR and an update—to the SR—for an overview.
    A de novo SR or an update of an SR—having access to primary study-level data—is best suited for quantitative combination of findings through meta-analyses (MAs), indirect treatment comparisons (ITCs), or network meta-analyses (NMAs) to provide a summary measure or answer a question about which intervention works best or is the safest.
    With the SR as the unit of analysis, there are limitations in the analytic capabilities of an overview, and hence the research objectives it can fulfill. In an overview, quantitative combination of findings is more difficult, compared to narrative incorporation of findings, without going back to the primary study reports [15, 27]. This is especially true if relevant SRs are of low quality in methodology or reporting (leading to errors or missing data) or if they overlap significantly in their included primary studies (giving too much statistical power to certain studies) [15, 27]. Further, unless reviewers have thoroughly examined the transitivity assumption (i.e., that the studies making different direct comparisons are sufficiently similar in all respects other than the treatments being compared) and found it to be valid, which is very difficult to do using SR reports, ITCs, or NMAs should not be conducted in overviews [9, 15, 31, 34]. Therefore, an overview of SRs—relying on review-level data—is best suited for examining a body of SR evidence to provide overall trends in research findings or answer a question about which interventions are effective or safe or for exploring if and why the evidence base on a topic or question is heterogeneous [27]. ITCs and NMAs in overviews are explicitly discouraged, and informal indirect comparisons should also be avoided [9].
  *Consideration II: If the planned review requires quantitative combination of findings through MAs, ITCs, or NMAs to provide a summary measure or to rank interventions, a de novo SR or an update of an SR should be considered instead of an overview of SRs. In an overview, a narrative, not quantitative, synthesis should be conducted.*
II. State of the existing literature
  3. Relevance: Are there one or more relevant SRs available?
    To answer this question, database searches with appropriate SR filters or targeted searches for potentially relevant SRs may be needed. Potential SRs of interest could then be assessed on whether they meet eligibility criteria of the planned review, as specified by the PICO elements [28, 29]. If the scope of an SR is identical to or narrower than that of the planned review, the entire SR would be relevant. If its scope is broader, the subset of its findings that meets the eligibility criteria of the planned review would be relevant, if reviewers are able to extract the relevant results separately (e.g., from a subgroup analysis).
    If there is no relevant SR, conducting a de novo SR is likely necessary.
    For an update, there must be one or more relevant SRs available that each perfectly or almost perfectly covers the research question. It may be possible to integrate multiple SRs in an update [15, 19], but this is discouraged due to logistical and methodological complexities, as it would likely require merging existing SRs first with each other and secondly with newly identified primary studies [5, 9]. Therefore, our recommendation is to handle multiple relevant SRs through either an overview of two or more SRs or an update of a single SR that is “best” in terms of relevance, quality, comprehensiveness, and currency [18].
    For an overview, there should be two or more relevant SRs available that each covers at least one “part” of a research question, with all SRs combined resulting in perfect or almost perfect coverage. There is no accepted minimum or maximum number of SRs required for an overview, although recommendations range from including a minimum of “two or more” SRs [9] to “5–10 or more” SRs [29].
  *Consideration III: The quantity of relevant SRs available may rule out certain review types for the planned review. If there is no relevant SR, no overview or update could be conducted. If there is only one relevant SR, no overview should be conducted.*
  4. Methodological quality: Are the relevant SRs of sufficiently high quality in methodology?
    While it may sometimes be appropriate to include all relevant SRs in the planned review for completeness, it is often advised to assess the methodological quality of existing SRs and only use high-quality SRs in updates or overviews to build on high-quality literature [3, 6, 9, 15, 19, 28, 31]. In the absence of high-quality SRs, replication in the form of a de novo SR may be deemed appropriate to produce a higher-quality SR. [6, 17, 36]
    To assess methodological quality, relevant SRs should be critically appraised for their methodological rigour [17, 29]. Established critical appraisal tools, such as AMSTAR 2 [37] or ROBIS [38], can be used [17, 29]. There is no broadly accepted threshold for what is considered sufficiently high quality in methodology for a relevant SR to be used in a planned review, although there is guidance for what is good, fair, or poor quality [3, 18]. The threshold for sufficiently high quality in methodology may vary depending on the purpose of the review, how the relevant SRs will be used, whether there are high-quality SRs available, and what resources are available for the planned review. Nevertheless, criteria for determining whether relevant SRs are of sufficiently high quality in methodology should be established a priori for each planned review to minimize bias [28].
  5. Reporting quality: Are the relevant SRs of sufficiently high quality in reporting?
    Poor reporting in existing SRs would make it difficult not only to extract data but also to decide whether the SRs are relevant, of high methodological quality, comprehensive, and up to date, and hence, useful for the planned review. Therefore, high quality in reporting is desirable [15, 19], especially if the SRs are to be used in overviews without going back to primary study reports.
    To assess quality in reporting, reporting guidelines may be used [32], supplemented with additional criteria required for the planned review (e.g., exact data needed, such as point estimates and associated variability in a specific format). Available reporting guidelines include PRISMA Statement (for all SRs) [39], SWiM (for SRs with narrative syntheses) [40], and MOOSE (for SRs with MAs of observational studies in epidemiology) [41]. There is no broadly accepted threshold for what is considered sufficiently high quality in reporting for an existing SR to be used. However, at the least, it should be well written, describe the PICO elements and methods clearly, and report all necessary data in an easily extractable form.
  *Consideration IV: Ideally, only SRs of high quality in both methodology and reporting should be used for updates and, in many cases, for overviews. In the absence of high-quality SRs, replication in the form of a de novo SR may be appropriate.*
  6. Comprehensiveness: Are the relevant SRs comprehensive?
    To answer this question, relevant SRs should be examined on whether they include all relevant PICO elements of the planned review either individually (i.e., a single SR is comprehensive on its own) or together (i.e., two or more SRs are comprehensive when combined). If they do not, using them in an overview may result in research gaps in the final review, as it is not recommended that an overview of SRs be supplemented with primary studies due to logistical and methodological complexities [5, 9]. Therefore, a research question that cannot be fully addressed with existing SRs due to important research gaps in those SRs may be best addressed by a de novo SR or an update of an SR (e.g., with a search for additional data from an expansion of one or more of the PICO elements or a re-analysis of the available data for a different outcome) if primary studies are available to fill those gaps. On the other hand, if two or more relevant SRs are available that when combined result in perfect or almost perfect coverage [2, 9, 10, 27, 32–35], an overview of SRs may be appropriate.
  7. Currency: Are the relevant SRs up to date?
    In general, an SR may be considered to be up to date when (1) there is no new evidence or (2) there is new information, but it is unlikely to change the review conclusions [34]. However, the definition of currency varies from one field to another, as the acceptable time period from the last date of search will depend on various factors, such as the activity or advances in the field or in methods, the importance and urgency of the research question being addressed, and the level of uncertainty around the evidence base [3, 8, 19, 42]. Criteria for determining the currency or outdatedness of relevant SRs need to be established a priori for each planned review to minimize bias [28, 43].
    For those overviews that have the objective to answer a research question about intervention effects (as opposed to the objective to describe a body of literature), all relevant SRs should be sufficiently up to date [9]. Whether an SR is sufficiently up to date should be determined with the help of experts in the field of research who have the knowledge to speak to the various factors that define currency mentioned above. Therefore, a research question that cannot be fully addressed with existing SRs because they are out of date may be best addressed by an update of an SR—if there is a high-quality SR available—or by a de novo SR—if there is no high-quality SR available—that is, if new evidence is available to bring it up to date [3].
  8. New evidence: Are there additional relevant primary studies missing from the relevant SRs?
    Additional primary studies that are relevant to the research question but not captured by relevant SRs may be identified during scoping or through expert input. They may be (1) newly published studies or (2) studies not identified by relevant SRs because the SRs had narrower scopes, searched fewer databases, or had limited information sources (e.g., no grey literature). Since an overview of SRs should not be supplemented with primary studies [5, 9], a research question that cannot be fully addressed with existing SRs because additional primary studies would be needed for comprehensiveness or currency may be best addressed by an update of an SR—if there is a high-quality SR available—or by a de novo SR—if there is no high-quality SR available.
  *Consideration V: If relevant SRs are not comprehensive or are outdated and need to be supplemented with additional primary studies that are available, an overview of SRs may not be appropriate. Instead, an update of an SR—if there is a high-quality SR available—or a de novo SR—if there is no high-quality SR available—is recommended for consistency in the analytic approach at the study level.*
  9. Discordance in results: Are the findings of the relevant SRs discordant for unknown reasons?
    If there are two or more relevant SRs addressing the same research question with matching PICO elements, and if they are of sufficiently high quality in both methodology and reporting, their results could then be compared. Results are concordant if they match with respect to the direction, magnitude, and statistical significance of the estimated intervention effects for the outcome(s) of interest and in terms of the interpretations and inferences made by the SR authors [44]. Conversely, results are discordant if they differ in any of those aspects [44]. Such differences may or may not be important enough to lead to different health care decisions [44].
    If the results of relevant SRs with matching PICO elements are discordant and if the differences matter for the planned review, reasons for discordance should be explored [15, 18, 19, 30, 35, 44]. Potential reasons for discordance include differences in the methods or timing of different SRs that lead to different sets of studies being included, data being extracted, analyses being conducted, or syntheses or interpretations being made [44]. If reasons for discordant findings are clear, and if there is certainty around which discrepant results to trust [15, 30, 35]—likely those of high quality in both methodology and reporting and most up to date—reviewers may choose one or more SRs that are most appropriate for the planned review and conduct an update or an overview. If reasons for discordant findings are unclear and if there is uncertainty around which discrepant results to trust [15, 30, 35], a de novo SR may be indicated, instead of using one or more of the conflicting SRs [15, 19].
  *Consideration IV: If the results of two or more relevant SRs with matching PICO elements are discordant, and reasons for discordance cannot be reliably determined, a de novo SR may be needed.*

### Part 2. Making the decision

**Fig. 1:**
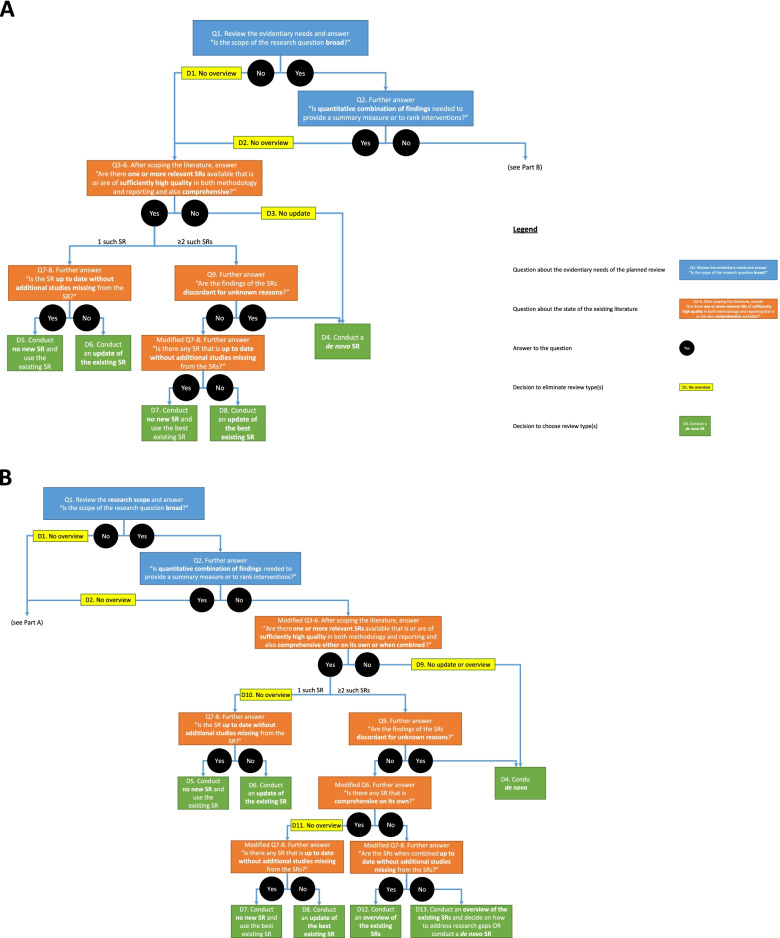
Flowchart for choosing one review type over another

A flowchart is presented in Fig. 1 and described below. This flowchart depicts the nine key factors and six considerations outlined above in the order that we see as helpful for selecting a specific review type to conduct for evaluating intervention effects in an HTA. The flowchart should be followed from top to bottom, first with Part A and then with Part B. Each question is indicated with a “Q” followed by a number that corresponds to the numbering of the key factors identified above. Both Part A and Part B begin with Q1 and Q2 (in blue boxes), which address the evidentiary needs of the planned review, and then proceed to Q3 through Q9 (in orange boxes), which address the state of the existing literature. Some of the questions from Q3 to Q9 are combined or modified for further investigation. Depending on the responses to the questions (“yes” or “no” in black circles), some review types are eliminated through this decision-making process (in yellow boxes), leading to one decision at the end (in green boxes).

If there is only a single relevant SR that is high quality in both methodology and reporting, comprehensive, and up to date, there is likely no need for a new SR of any kind for the specific research question (see D5 in Fig. 1 in both Part A and Part B). In this case, the existing SR can and should be used instead of conducting a new SR. Similarly, if there are multiple such SRs without discordant results for unknown reasons, no new SR should be conducted, and the best existing SR can be used (see D7 in Fig. 1 in both Part A and Part B). In all other cases, a decision on which review type to conduct must be made.

For some situations, there are considerations regarding the key factors identified above that are absolutely deterministic in ruling out certain review types, including the following:

- If the planned review requires quantitative combination of findings through MAs, ITCs, or NMAs, an overview should not be conducted (Consideration II; see D2 in Part A of Fig. 1).
- If there is no relevant SR, an update or an overview could not be conducted (Consideration III; see D3 in Part A and D9 in Part B of Fig. 1).
- If there is only one relevant SR, an overview should not be conducted (Consideration III; see D10 in Part B of Fig. 1).

For most situations, however, it is the interplay of multiple considerations regarding the key factors that informs the decision on which review type to conduct. In these cases, while no review types can be completely ruled out, one review type may be favoured over the others, as follows:

- If the planned review has a narrow scope (Consideration I), instead of an overview (see D1 in Part A of Fig. 1), it may be more appropriate to conduct a de novo SR (see D4 in Part A of Fig. 1) or an update of an SR (see D6 and D8 in Part A of Fig. 1), depending on whether one or more relevant SRs of high quality are available (Consideration IV) without discordant results for unknown reasons (Consideration VI). For example, if there is a single “best” relevant SR that is high quality in both methodology and reporting and comprehensive but needs an update with newly available data, new methods, or new analyses, with concordant results with other relevant SRs, an update may be the most appropriate review type. If there is no relevant SR at all or no relevant SR that is high quality in both methodology and reporting and comprehensive, a de novo SR may be needed.
- Conversely, if the planned review has a broader scope (Consideration I), and there are two or more relevant SRs available (Consideration III), an overview may be the most appropriate review type, especially if the relevant SRs are high quality in both methodology and reporting, comprehensive when combined, up to date, and concordant in their findings, with no need to be supplemented with additional primary studies (Considerations IV, V, and VI; see D12 in Part B of Fig. 1). If one or more of these conditions on the existing literature are not met, reviewers should carefully consider whether an overview of SRs is the most appropriate review type. It may be more appropriate to conduct a de novo SR, especially if the number of relevant primary studies is manageable with given resources (see D4 and D13 in Part B of Fig. 1), or an update if there is a single “best” relevant SR that is high quality in both methodology and reporting and comprehensive on its own (see D 6 and D8 in Part B of Fig. 1).

The flowchart in Fig. 1 outlines a decision-making process for selecting a review type to conduct, capturing all of the considerations from above.

## Discussion

This paper shares guidance that can be used when selecting the most appropriate review type to conduct when evaluating intervention effects in an HTA. It identified nine key factors and six considerations concerning the evidentiary needs of the planned review and the state of the existing literature. Those factors and considerations were then organized into a summary and a corresponding flowchart that can be used as a decision tool, balancing potential benefits of leveraging existing SRs against any drawbacks of not starting a new SR from scratch. With that, a reviewer may choose to conduct no new SR at all, update an existing SR, use multiple existing SRs in an overview, or conduct a de novo SR. This guidance is aligned with the literature from various organizations [3, 6, 9, 15, 18, 19, 27–31] and other research groups [2, 8, 10, 32–35, 45], including a recent publication that highlights eliminating research waste by avoiding unnecessary duplication of SRs [17].

To fully contemplate these key factors and considerations for a planned review, there must be a clear research question as well as a good understanding of the quantity, quality, and other characteristics of relevant SRs and primary studies that are available. Therefore, this approach may require detailed scoping and perhaps a process change as well as additional resources as an “upfront” investment in the conduct of an HTA. This challenges the notion that leveraging existing SRs necessarily saves resources, and it reinforces the idea that existing SRs should be leveraged for other reasons, such as summarizing the existing literature at higher levels and avoiding or minimizing duplication or redundancy in research, all of which had been raised by other groups as well [15, 17, 19, 46]. Further, any additional work performed to follow the guidance presented here and apply the decision tool need not be wasted, as it could identify existing SRs that fully meet the needs of the planned review and actually free up and save resources or it could be used during the review process, regardless of the review type chosen.

The decision tool presented here depicts general guidance and should be contextualized to each case. For example, it might not always be clear if the research scope for the planned review is broad or narrow (Q1 in Fig. 1 in both Part A and Part B). Further, even for a narrow topic, if there are multiple relevant SRs, an overview may be considered as long as there is no need for quantitative combination of findings. In another example, if there is a high-quality SR that is almost but not fully comprehensive (Q3-6 in Fig. 1 in both Part A and Part B), the reviewer may decide to update that SR with revised PICO elements, instead of conducting a de novo SR, although this would generally be considered a purposeful replication and perhaps more of a de novo SR instead of an update in any case [6–8, 17]. Therefore, the decisions outlined in the tool should be viewed as examples in ideal situations instead of a collection of definitive and exhaustive possibilities, and going against those decisions is possible and may even be appropriate in certain cases. The flowchart should be seen as simply highlighting the many interacting considerations that need be contemplated during the decision-making process. We suspect that others who conduct evidence syntheses are also working through these decisions, perhaps on an ad hoc basis, and present this work to share our current decision-making process and invite commentary and reflections.

Regardless of which review type is used, it is essential that the project protocol and report provide a list of existing relevant SRs identified in the literature through the scoping activity (e.g., as an appendix) and the rationale behind selecting a specific review type over others for transparency purposes. During scoping, effort should be made to also identify ongoing SRs (e.g., through PROSPERO). If ongoing or planned reviews are identified, opportunities for brokering or collaborating or other ways to incorporate them into the decision-making process for selecting a review type should be explored.

We considered incorporating into the flowchart rapid approaches [12] and living approaches [13], either or both of which could be overlaid on each review type (e.g., rapid SR, rapid update, or rapid overview; living SR or living overview; living rapid SR or living rapid overview). A rapid approach alters, simplifies, or omits components of a systematic process to produce information in a shorter period of time [12]. This approach may involve searching fewer databases or engaging a single reviewer instead of dual reviewers and may be used under resource constraints (e.g., short timelines or limited funding) [20], especially if there is already some high-quality evidence with one or more relevant SRs available and no unexplained discrepant findings across the SRs. A living approach continually updates an SR or an overview, incorporating relevant new evidence as it becomes available [13]. This approach may be used if a priority research question cannot be fully addressed because there is an important level of uncertainty in the existing evidence and if new evidence that will impact the current conclusions is likely to emerge soon in a research field that is moving relatively quickly [43]. As the literature on these approaches is still actively emerging, we did not incorporate these review approaches into the flowchart but identify them as potential additional considerations, as also noted by others [17]. In other words, reviewers could use the flowchart provided here to first select the most appropriate review type to conduct and then decide to apply rapid and/or living approaches to the chosen review type as needed.

## Conclusions

This work provides guidance on how to balance a myriad of factors and considerations to help select the most appropriate review type to conduct when evaluating intervention effects in an HTA. The decision-making process presented is undoubtedly complicated, yet fundamental for every review, given the exponential growth in the number of published SRs [14, 15] and high-resource requirements for completing new SRs [15]. While leveraging existing SRs may or may not save resources, doing so will likely contribute to a better synthesis of the existing literature and a reduction of research waste and therefore lead to a more efficient use of resources in HTAs.

We hope this guidance adds clarity to the many competing considerations when deciding which review type to conduct and facilitates that decision-making process. As we continue to test and update this decision tool, we invite feedback from others who may have their own guidance or apply ours in their work. While we focused on SRs of intervention effects, the application of this guidance to other types of reviews, such as qualitative or mixed methods reviews, should be explored in the future.

## Data Availability

Not applicable.

## Appendix 1

Table 2

**Table 2:** Database search strategy

**OVERVIEW**	
Interface:	Ovid
Databases:	MEDLINE All (1946-present)
Date of Search:	March 5, 2019
Alerts:	Monthly search updates until manuscript submission
Study Types:	No filters were applied to limit the retrieval by study type
Limits:	Publication date limit: none Language limit: English
**SYNTAX GUIDE**	
/	At the end of a phrase, searches the phrase as a subject heading
MeSH	Medical Subject Heading
exp	Explode a subject heading
*	Before a word, indicates that the marked subject heading is a primary topic; or, after a word, a truncation symbol (wildcard) to retrieve plurals or varying endings
?	Truncation symbol for one or no characters only
adj#	Requires terms to be adjacent to each other within # number of words (in any order)
.ti	Title
.ab	Abstract
.hw	Heading word; usually includes subject headings and controlled vocabulary
.kf	Author keyword heading word
**MEDLINE STRATEGY**	
**#**	**Searches**
1	((updating or existing systematic review* or “out-of-date” or outdated or up-to-dateness or redundant or redundancy or incorporating or overlap* or choosing or selecting or deciding or integrating or discordan* or concordan* or replicat*) and (systematic review* or comparative effectiveness review* or overviews of reviews or overview of reviews or Cochrane review* or meta-analyses)).ti,kf.
2	(de novo adj3 review*).ti,ab,kf.
3	(overview of reviews or overviews of reviews or overview of systematic reviews or overviews of systematic reviews).ti,ab,kf.
4	(overview of overviews or overviews of overviews).ti,ab,kf.
5	((updat* adj2 review*) or (updat* adj2 systematic review*) or (updat* adj2 comparative effectiveness review*)).ti,ab,kf.
6	review design.ti,ab,kf.
7	(existing review* or existing systematic review* or existing comparative effectiveness review*).ti,ab,kf.
8	(umbrella review* or umbrella systematic review*).ti,ab,kf.
9	(review of reviews or reviews of reviews or review of systematic reviews or reviews of systematic reviews).ti,ab,kf.
10	(incorporating adj3 review*).ti,ab,kf.
11	multiple systematic review*.ti,ab,kf.
12	(synthes?s adj4 review*).ti,ab,kf.
13	secondary evidence.ti,ab,kf.
14	(previous review* or previous systematic review*).ti,ab,kf.
15	(overlap* adj3 review*).ti,ab,kf.
16	(conduct* adj3 (overviews of reviews or systematic review* or comparative effectiveness review*)).ti,kf.
17	or/2-16
18	(guidance or methodolog* or consensus).ti,hw,kf. or *meta-analysis as topic/ or *review literature as topic/
19	17 and 18
20	((choos* or select* or appropriate* or decid* or decision* or guidance or methodology*) adj4 (review design or review type)).ti,ab,kf.
21	(updating review* or updating systematic review* or updating comparative effectiveness review*).ti,ab,kf.
22	(integrating review* or integrating systematic review* or integrating comparative effectiveness review*).ti,ab,kf.
23	(de novo review* or de novo systematic review*).ti,ab,kf.
24	(discordant review* or discordant systematic review* or discordant comparative effectiveness review*).ti,ab,kf.
25	(replicat* adj3 systematic review*).ti,ab,kf.
26	up-to-date systematic reviews.ti,ab,kf.
27	when is an update an update.ti.
28	or/20-27
29	1 or 19 or 28
30	limit 29 to english language
31	remove duplicates from 30

## Appendix 2

Table 3

**Table 3:** Grey literature search strategy

Search dates:	March 7-8, 2019
Keywords:	Systematic reviews / health technology assessment / HTA Updating / de novo / existing / umbrella methodology / guidance
Limits: Updated:	Publication years: no limit; English language only March 3-5, 2021

Relevant websites from the following sections of the CADTH grey literature checklist *Grey Matters: A Practical Tool for Searching Health-Related Grey Literature* (https://www.cadth.ca/grey-matters) were searched:

- Health Technology Assessment Agencies
- Databases (free)
- Internet Search

## Appendix 3

Table 4

**Table 4:** Excerpts of relevant text from the literature review

Author	Review Type	Scope	Objective	Analytic Approach	Existing Literature	Notes
**SR or HTA guidelines**						
Joanna Briggs Institute [27]	Umbrella review	“Conduct of an Umbrella Review offers the possibility of addressing a broad scope of issues related to a topic of interest.” “The principal reason is to summarize evidence from many research syntheses (Becker and Oxman 2011). These may include analyses of evidence of different interventions for the same problem or condition, or evidence from more than one research synthesis investigating the same intervention and condition but addressing and reporting on different outcomes. Similarly, a researcher or reviewer may wish to summarize more than one research synthesis for different conditions, problems or populations.”	“The wide picture obtainable from the conduct of an Umbrella Review is also ideal in highlighting if the evidence base around a topic or question is consistent or if contradictory or discrepant findings exist, and in exploring and detailing the reasons why. Investigation of the evidence with an Umbrella Review allows assessment and consideration of whether reviewers addressing similar review questions independently observe similar results and arrive at generally similar conclusions.”	“…a systematic review is the main and often sole “study type” that is considered for inclusion (Becker and Oxman 2011; Hartling et al. 2012; Smith et al, 2011).” “The principle focus of a JBI Umbrella Review is to provide a summary of existing research syntheses related to a given topic or question and not to re-synthesize, for example, the results of existing reviews or syntheses with meta-analysis or meta-synthesis.”	“…if current, multiple, good quality, systematic reviews exist about a given topic or question, any reviewer should reconsider the need to conduct yet another review addressing the same issue. Rather, these may be the basis to conduct an Umbrella Review and summarize or synthesize the findings of systematic reviews already available.”	None.
Cochrane Collaboration [9]	Overview	“Cochrane Overviews can address five different types of questions related to healthcare interventions. Specifically, they can summarize evidence from two or more systematic reviews: • of different interventions for the same condition or population; • that address different approaches to applying the same intervention for the same condition or population; • of the same intervention for different conditions or populations; • about adverse effects of an intervention for one or more conditions or populations; or • of the same intervention for the same condition or population, where different outcomes or time points are addressed in different systematic reviews.” “The primary reason for conducting Cochrane Overviews is that using systematic reviews as the unit of searching, inclusion, and data analysis allows authors to address research questions that are broader in scope than those examined in individual systematic reviews…a second reason for conducting a Cochrane Overview is that they may be associated with time and resource savings, since the component systematic reviews have already been conducted. A third reason for conducting a Cochrane Overview is in cases where it is important to understand the diversity present in the extant systematic review literature.” “…it is preferable to conduct a Cochrane Review of interventions if authors anticipate the need to conduct searches for primary studies (i.e., many relevant primary studies are not included in systematic reviews) or to extract data directly from primary studies (i.e., the anticipated analyses cannot be conducted on the basis of information provided in the systematic reviews). Using primary studies as the unit of searching, inclusion and data analysis allows authors to extract all data of interest directly from the primary studies and to report these data in a standardized way. It is also preferable to conduct a Cochrane Review of interventions if authors wish to conduct network meta-analyses, which allow authors to rank order interventions and determine which work ‘best’.”	“Overviews can describe the current body of systematic review evidence on a topic of interest, or they can address a new review question that wasn’t a focus in the included systematic reviews.” “There are several instances where authors should not conduct a Cochrane Overview. Overviews do not aim to: • repeat or update the searches or eligibility assessment of the included systematic reviews; • conduct a study-level search for primary studies not included in any systematic review; • conduct a new systematic review within the Overview; • use systematic reviews as a starting point to locate relevant studies with the intent of then extracting and analysing data from the primary studies (this would be considered a systematic review, or an update of a systematic review, and not an Overview); • search for and include narrative reviews, textbook chapters, government reports, clinical practice guidelines, or any other summary reports that do not meet their pre-defined definition of a systematic review; • extract and present just the conclusions of the included systematic reviews (instead, actual outcome data—narratively reported study-level data and/or meta-analysed data—should be extracted and analysed, and Overview authors are encouraged to interpret these outcome data themselves, in light of the Overview’s research questions and objectives); • present detailed outcome data for primary studies not included in any included systematic review; or • conduct network meta-analyses…”	“…the unit of searching, inclusion and data analysis is the systematic review rather than the primary study.” “Although Overviews often present evidence from two or more systematic reviews of different interventions for the same condition or population, they should rarely be used to draw inferences about the comparative effectiveness of multiple interventions. This means that they should not directly compare interventions that have been examined in different systematic reviews with the intent of determining which intervention works ‘best’ or which intervention is ‘safest’.” “We discourage indirect comparisons in Overviews. This is especially relevant for authors conducting Overviews that examine multiple interventions for the same condition or population; it is also relevant for authors regardless of whether the systematic reviews included in the Overview present their data using meta-analysis or simple narrative summaries of results. The reason is that the assumption underlying indirect comparison—the transitivity assumption—can rarely be assessed using only the information provided in the systematic reviews…” “Overviews that examine multiple interventions for the same condition or population will often juxtapose data from different systematic reviews. Sometimes, these data appear in the same table or figure. Overviews that present data in this way can inadvertently encourage readers to make their own indirect comparisons. In cases where Overviews may facilitate inappropriate informal indirect comparisons, Overview authors must avoid ‘comparing’ across systematic reviews.”	“Overview authors should recognize that there will be some heterogeneity in the included systematic reviews and should consider whether or not the extent and nature of the heterogeneity precludes the utility of the Overview. Authors may find it helpful to consider whether: • the systematic reviews are, or are likely to be, sufficiently up-to-date; • the systematic reviews are, or are likely to be, sufficiently homogeneous in terms of their populations, interventions, comparators, and/or outcome measures (i.e., such that it would make sense from the end-user’s perspective that the individual systematic reviews were presented in a single product); • the systematic reviews are, or are likely to be, sufficiently homogeneous in terms of what and how outcome data are presented (such that they provide a useful resource for healthcare decision making); • the amount and type of outcome data presented is, or is likely to be, sufficient to inform the Overview’s research question and/or objectives; and • the systematic reviews are, or are likely to be, of sufficiently low risk of bias or high methodological quality (i.e., authors should have reasonable confidence that results can be believed or that estimates of effect are near the true values for outcomes…”	None.
Cochrane Collaboration [6]	Update	Not reported.	Not reported.	Not reported.	“When deciding whether to update a particular review, the first consideration should be to determine whether the review question remains relevant to decision makers, and is well-targeted to answer current questions in policy and practice. Knowledge of the particular field will be required to answer this question. Checking whether the existing review is frequently accessed or cited can also be useful to indicate whether there is a need to update. A second aspect to this question is whether the original review was conducted well and used appropriate methods (Garner et al 2016). If the review question remains fundamentally of interest, additions and improvements may be possible to enhance the review’s methods (see Section IV.3.4). Depending on the changes required, it may be more appropriate to conduct a new review from scratch meeting current standards. A comparison between currently recommended methods and the methods used in the review can identify any important changes required.”	None.
Agency for Healthcare Research and Quality [28]	Integration of existing systematic reviews into new reviews	Not reported.	Not reported.	Not reported.	“Although it is important for systematic reviewers to consider all potentially relevant primary studies, it may not be necessary that all potentially relevant prior systematic reviews be considered. It is more important to assess and include prior reviews that are most relevant and of high quality than to attempt to include all reviews. Several factors can be considered in assessing relevance, including the date(s) of the search (currency), and review methods. Relevancy should be assessed using the PICOT (population, intervention, comparison, outcome, time) framework.”	None.
Agency for Healthcare Research and Quality [15]	Integration of existing systematic reviews into new reviews	“Some described that including existing reviews sometimes enables them to cover a wider range of questions and elements of questions (as denoted by PICOTS) when existing systematic reviews address important aspects of new review key questions.”	Not reported.	“When incorporating existing reviews into a new or updated review, EPC members most often described qualitative or narrative incorporation of the existing reviews, noting that quantitative combination of findings (without going back to all primary studies) is more difficult and potentially introduces error, and thus, is less commonly done.”	“Methods by which relevant existing systematic reviews can be evaluated for quality of methodological approach, using AMSTAR or other commonly used tools, with a focus on potentially incorporating only reviews meeting certain quality criteria into the proposed review…” “there are other instances when it would not make sense to use an existing review, such as when there are few studies on a given topic or when there are multiple conflicting systematic reviews.” “If more than one high-quality review is found with discordant findings, it may be an indication to start a de novo review on that key question.”	“Overall themes were identified from the discussions with EPCs: • EPCs most commonly used existing reviews as a source of relevant literature and as context for the introduction or discussion section of reviews. • Existing reviews were most useful when key questions and/or PICOTS-SD (population, intervention, comparator, outcome, time frame, setting, and study design) matched or when they addressed a specific subquestion of the new review. • Using existing reviews was often more resource intensive than completing a review from scratch. • EPCs expressed that they often did not trust aspects of reviews conducted by others. • When relevant and rigorous, incorporating prior reviews into the review being undertaken by the EPC was clearly valuable in at least two instances: 1) allowing larger scope of the review being undertaken without additional resources, or 2) providing summarized evidence when a new in depth review of primary literature would not be feasible (for example, existing reviews provide individual patient data analysis or include hundreds of trials, supplemented by author-provided data).”
Belgian Health Care Knowledge Centre [29]	Meta-review/ review of reviews	Not reported.	Not reported.	Not reported.	“The main factors that have to be taken into account when deciding to perform a meta-review are the amount of reviews available on the topic and the time available for doing a review. In general a shortage in time and/or a high number of reviews available, may favour the decision to do a meta-review.” “No general guidelines exist but from a pragmatic point of view it is advisable to discuss in the research team if a meta-review is a viable option, if there are approximately 5-10 or more recent reviews fairly matching your research question.” “Other factors to consider in the decision: • Applicability of published reviews to your topic: are the reviews really about the intervention you are interested in and did they extract data on the outcome of your interest?; • Recency of the reviews, especially the search date is essential: it is good to take the search of one of the reviews and rerun it to see how many recent primary studies will be missing and to estimate if these could possibly alter the review conclusions; • Methodological quality of reviews: are the reviews well performed? Hereto an assessment tool can be applied, e.g. AMSTAR85 as also proposed in the general KCE process notes (other assessment instruments exist and an overview is presented in among others Zeng et al. (2015) and Pieper et al. (2014); • Number of included studies in reviews and overlap between the reviews: if most or all (recent) reviews only include one or two studies, it has probably not much sense to do a new review, but if each review includes tens of primary studies with only a small overlap, this pleas for a new review; • Availability of meta-reviews: it can well be the case that on popular topics, one or more meta-reviews already exist, and if so the decision to do a new one should be weighted with the same factors as mentioned above. It can also be considered to do a review of meta-reviews, but in general this is not advised because the primary evidence is getting out of sight. No clear-cut decision process can be presented here; it really needs discussion in the research team. There are many trade-offs in determining whether it is more efficient, and a methodologically sound process to rely on a prior review or to start from scratch. And keep in mind that a meta-review can only be as good as the reviews and primary studies on which it is based.”	“There are pros and cons for this approach. Main assumed advantage of doing a meta-review is time saving since someone else already searched, sifted, assessed and analysed the available evidence from primary studies. On the other hand reviews are on a higher abstraction level than primary studies and it may be difficult to get grasp on what really happened and how it was studied; details are lost. Moreover, when performing a meta-review, you are dependent on the intervention and outcome criteria that were formulated by others and these may be slightly different from what you want for your research question.”
World Health Organization [30]	Update	Not reported.	Not reported.	Not reported.	“It is not always necessary to commission a new systematic review (see Fig. 8.1). If one or more relevant, current and high-quality systematic reviews exist, these should be used. Updates, if needed, are usually less expensive and time-consuming than new reviews.” “…updating an existing systematic review is a reasonable option provided a high quality, fully reported review exists that uses current methods for identifying, appraising and synthesizing the evidence.”	“To assess relevance, compare the key question of the existing systematic review to the key questions that were developed during the guideline scoping exercise, considering each component of PICO. Most frequently, the existing review does not entirely match the current key question. Nonetheless, the review may address one facet of WHO’s key question or provide useful background information, and the list of included studies may inform WHO’s systematic review. If the existing systematic review addresses one of the key questions of the guideline, then its quality should be assessed. The following checklists may be used to assess the quality of systematic reviews: • assessment of multiple systematic reviews (AMSTAR) (11), or R-AMSTAR (12); • Oxman and Guyatt index for the quality of review articles (1991) (13). “Once a systematic review has been found relevant and of high quality, whether it is up to date or not must be determined. There is no rule for dismissing a review on the basis of the time since publication (for example, two years); it depends on the topic and on the availability and rate of production of new information.”
Agency for Healthcare Research and Quality [18]	Replacing de novo processes using existing systematic reviews	Not reported.	Not reported.	Not reported.	“If the selected relevant, high-quality SRs have discordant findings, EPCs should explore the reasons for these disagreements. If EPCs cannot readily give reasons for the discordant findings, then they can regard this as an indication that they need to adopt a de novo approach to answer that key question.” “If EPCs find that several recent, relevant, and high-quality SRs are appropriate for a given CER, they then need to determine how best to proceed. One approach is to incorporate the single “best” existing SR (most relevant and least biased) into their own reports [6]. However, selecting a single review may pose the risk of introducing selection bias; EPCs must ensure transparency in their criteria for eligibility. Another approach is to conduct a meta-review (also known as an “umbrella review”), whereby they select all relevant, high-quality SRs that meet an a priori publication date threshold and then assess the consistency among them [26, 27].”	“In general, good-quality SRs should be defined as those that have few or no methodological shortcomings and a low risk of bias. Fair-quality SRs have some methodological flaws but the EPC conducting the CER determined that the flaws will not seriously bias or invalidate the results. Poor-quality SRs contain a serious flaw or flaws that, in the judgment of the EPC conducting the CER, are highly likely to bias or invalidate the results.”
Agency for Healthcare Research and Quality [19]	Using existing systematic reviews	Not reported.	Not reported.	Not reported.	“…reviews should be excluded when the number of primary studies is small and any gains in efficiency by using the reviews would be minimal at best.” “Systematic reviews should be as free of bias as possible if they are to reliably inform guideline recommendations or other policy decisions (2). Quality assessment of existing systematic reviews is therefore a critical step and should address both the methods used by the systematic reviewers to minimize bias as well as the transparency and completeness of reporting of review methods, individual study details, and results. In fact, the priority should be to include existing systematic reviews adhering to high methodological standards, rather than to routinely include all existing systematic reviews in order to be comprehensive.”	“…when higher-quality reviews disagree, a new independent review that carefully addresses potential sources of disagreement with careful sensitivity or subgroup analyses may be the most informative strategy.”
Recommendations from 8 HTA agencies* [31] *The 8 HTA agencies were the Agency for Quality and Accreditation in Health Care (AAZ, Croatia), Centre for Reviews and Dissemination (CRD, UK), Gesundheit Österreich GmbH (GÖEG/BIQG, Austria), Health Care Improvement Scotland (HIS, UK), Institute for Quality and Efficiency in Health Care (IQWiG, Germany), Belgian Federal Health Care Knowledge Centre (KCE, Belgium), Ludwig Boltzmann Institute for Health Technology Assessment (LBI-HTA, Austria), and National Institute for Clinical Excellence (NICE, UK).	Overview	Not reported.	Not reported.	Seven of the HTA agencies were open to searching for primary studies. “According to two HTA agencies, a narrative synthesis instead of a quantitative synthesis should be performed, while the third agency only notes that an evidence synthesis is likely to be problematic.” “In the context of overviews, a meta-analysis of meta-analyses should turn out to be difficult as some of the primary studies will usually be included in more than one meta-analysis. Pooling results would give too much statistical power to multiple included primary studies (Smith et al., 2011). Other ways of presenting findings will have to maintain a reasonable balance between complexity and a potential loss of important findings from the reviews.”	“Five of the HTA agencies have prerequisites for the conduct of overviews (Table 1). All of them state that relevant SRs must be of high quality, although only one HTA agency concretizes this by setting cut-off points for the review quality, based on the assessment with a critical appraisal tool.”	“Overviews should be updated when the corresponding reviews have been updated.”
Centre for Reviews and Dissemination at the University of York [3]	Update	Not reported.	Not reported.	Not reported.	“If an existing review is identified which addresses the question of interest, then the review should be assessed to determine whether it is of sufficient quality to guide policy and practice. In general, a good review should focus on a well-defined question and use appropriate methods. A comprehensive search should have been carried out, clear and appropriate criteria used to select or reject studies, and the process of assessing study quality, extracting and synthesizing data should have been unbiased, reproducible and transparent. If these processes are not well-documented, confidence in results and inferences is weakened. The review should report the results of all included studies clearly, highlighting any similarities or differences between studies, and exploring the reasons for any variations.” “If a high quality review is located, but was completed some time ago, then an update of the review may be justified. Current relevance will need to be assessed and is particularly important in fields where the research is rapidly evolving. Where appropriate, collaboration with the original research team may assist in the update process by providing access to the data they used. However, little research has been conducted on when and how to update systematic reviews and the feasibility and efficiency of the identified approaches is uncertain [11]. If a review is of adequate quality and still relevant, there may be no need to undertake another systematic review.”	None.
	Review of reviews	“Reviews of reviews are likely to be helpful when a review question is very broad and a number of systematic reviews have already been conducted in the topic area.”	Not reported.	Not reported.	“Reviews of reviews are likely to be helpful when a review question is very broad and a number of systematic reviews have already been conducted in the topic area.”	None.
**Academic papers**						
Tugwell et al. [17]	Replication, defined as: • “Direct replication by purposeful repetition to verify the findings of the original research question; or • Conceptual replication by purposeful broadening or narrowing of the research question in existing systematic reviews (e.g., across broader or more focused populations, intervention types, settings, outcomes, or study designs)”	Not reported.	“The need for replication of systematic reviews arises from concerns or lack of clarity about the technical or statistical methods or the judgments made, such as the subjective decisions related to defining criteria for inclusion of the population, intervention or exposure, and outcomes of interest; and data collection, synthesis, and interpretation [8].”	Not reported.	“Question 2. Is it likely that direct replication by repetition or conceptual replication by broadening or narrowing of the scope will address uncertainties, controversies, or the need for additional evidence related to: 2.1. The framing of the question in previous reviews? 2.2. The conduct and reporting of previous reviews? 2.3. Author influence or conflicts of interest in previous reviews? 2.4. Discordant findings in previous reviews?”	The authors included a replication comparative worksheet for appraising the body of evidence that can include multiple systematic reviews.
Noble et al. [2]	Systematic review	“…to address a highly focused clinical question.”	Not reported.	“Evaluates and summarizes the findings of all relevant individual studies, and if appropriate, combines the results of several studies to provide more reliable results.”	Not reported.	“The ‘gold standard’ of reviews because the review is based on explicit, prespecified and reproducible methods used to systematically search all sources of evidence and critically appraise, summarize and synthesize research findings…”
	Review of reviews/ umbrella review	“Useful when a review question is very broad and a number of systematic reviews have already been conducted in the topic area.”	Not reported.	“Compiles evidence from multiple research syntheses in order to summarize existing evidence and like systematic reviews follow clear methods.”	“Useful when a review question is very broad and a number of systematic reviews have already been conducted in the topic area.”	“…the different inclusion criteria adopted by the reviews included can make interpretation problematic.”
Ballard et al. [32]	Overview	“Overviews facilitate these broad comparisons…” “Overviews examine: (1) different interventions for the same condition or population (e.g. Jones et al., 2012), (2) the same intervention for different conditions or populations (e.g. Steultjens et al., 2005), (3) multiple outcomes of the same intervention for the same condition or population (e.g. Flodgren et al., 2011), or (4) adverse effects from the same intervention across multiple conditions (e.g. Singh Jasvinder et al., 2011).”	“…they serve the following three functions: 1. To identify gaps in the literature where multiple comparable studies may exist but a research synthesis has not been performed (Caird et al., 2015; Piso et al., 2015; Ryan et al., 2009; Cooper and Koenka, 2012; Santaguida et al., 2013) 2. To compare and contrast existing systematic reviews (Aromataris et al., 2014; Aromataris et al., 2015; Baker et al., 2014; Conn and Coon Sells, 2014; Cooper and Koenka, 2012; Pieper et al., 2014a; Pieper et al., 2012; Santaguida et al., 2013; Smith et al., 2011). 3. To provide a summary of evidence from existing systematic reviews, with or without synthesis (Becker and Oxman, 2011; Caird et al., 2015; Cochrane Comparing Multiple Interventions Methods Group, 2012; Cochrane Comparing Multiple Interventions Methods Group, 2013; Cooper and Koenka, 2012; Hartling et al., 2012; Hartling et al., 2014; Li et al., 2012; Pieper et al., 2014a; Pieper et al., 2012; Piso et al., 2015; Ryan et al., 2009; Smith et al., 2011; Thomson et al., 2013; Thomson et al., 2010).”	Not reported.	“As Pieper et al. (2012) point out, it is important to differentiate between methodological quality, which considers how well the systematic review was conducted, and reporting quality, which considers how well systematic reviewers have reported their methodology and findings. For this reason, it has been recommended that PRISMA be used in conjunction with a comprehensive, validated critical appraisal tool (Pieper et al., 2012; Shea et al., 2007; Oxman and Guyatt, 1991).”	None.
Lunny et al. [33]	Overview	“Overviews are typically broader in scope than systematic reviews (SRs) and may examine different interventions for the same condition, the same intervention for different conditions, or the same intervention for the same condition but focusing on different outcomes [5–8].”	“2.1 Define the purpose of the overview 2.1.1 Map the type and quantity of available evidence (e.g. types of interventions, outcomes, populations/settings, study designs but not effects) 2.1.2 Compare multiple interventions with the intent of drawing inferences about the comparative effectiveness of the interventions intervention for the same condition, problem or population 2.1.3 Summarize the effects of an intervention for the same condition, problem or population where different outcomes are addressed in different SRs 2.1.4 Summarize the effects of an intervention across conditions, problems or populations (e.g. “borrowing strength” when there is sparse data for a single condition and a similar mechanism of action for the intervention is predicted across conditions) 2.1.5 Summarize unexpected (including adverse) effects of an intervention across conditions, problems or populations 2.1.6 Identify and explore reasons for heterogeneity in the effects of an intervention (e.g. by examining reasons for discordant results or conclusions across SRs)”	Not reported.	Not reported.	None.
Garner et al. [8]	Update of a systematic review	“An update asks a similar question with regard to the participants, intervention, comparisons, and outcomes (PICO) and has similar objectives; thus it has similar inclusion criteria.”	Not reported.	Not reported.	Not reported.	“Decisions about whether and when to update a systematic review are judgments made at a point in time. They depend on the currency of the question asked, the need for updating to maintain credibility, the availability of new evidence, and whether new research or new methods will affect the findings.”
Hartling et al. [34]	Systematic review	“A limitation of SRs, as they have evolved within the healthcare field, is that often they are narrow in scope and may exclude competing interventions for a given condition. They often focus on direct pairwise comparisons and may lack formal comparisons across different interventions that are critical for informed decision making by end-users, including clinicians, policymakers and consumers.”	Not reported.	Not reported.	“The purpose of SRs is to collate relevant evidence from individual studies to answer a specific research question.”	None.
	Overview	“Overviews compile information from multiple SRs in order to provide a comprehensive synthesis of the evidence examining: different interventions for the same condition; different outcomes for the same intervention in the same condition; the same intervention for different conditions or populations; or adverse effects from the same intervention across multiple conditions (27).”	Not reported.	“The Cochrane Multiple Intervention Methods Group now emphasizes that overviews should not simply summarize SRs, rather they should integrate or synthesize the evidence (36).” “…conducting an NMA may be challenging within the context of an overview, given the previously mentioned inconsistencies among individual SRs.”	“Overviews compile data from multiple SRs…”	“Because an overview is based on existing SRs that have already identified the relevant studies and extracted data, carrying out an overview is more feasible and efficient than undertaking an SR.” “…the authors are dependent on the decisions and methods used within the relevant SRs.”
	Comparative effectiveness review	“In this article, we use the term ‘comparative effectiveness review (CER)’ to refer to a review that collates evidence from individual studies to describe the relative benefits (or harms) of a range of interventions. These reviews use explicit, systematic methods similar to those of SRs. **They may be considered SRs**; however, they are generally broader in scope than the focused pairwise comparisons typical of Cochrane SRs, the reason for distinguishing them in this article. Furthermore, they may be broader in terms of including both randomized and nonrandomized studies in order to identify measures of effectiveness and safety. They differ from overviews in that, for the most part, they focus on primary studies rather than SRs as the unit of analysis.”	Not reported.	Not reported.	Not reported.	None.
Cooper et al. [35]	Overview	“…overviews often seek to answer questions that are much broader in scope than questions that are typically asked by a single research synthesis.”	Not reported.	Not reported.	Not reported.	“If an overview is meant to replace the syntheses it covers, then it is even further removed from the research front.” “…overviewers would be well served to include a section in their report in which they at least reference and discuss what these recent advances are and how they relate to the work covered in the constituent research syntheses (see also Thomson et al., 2010).”
Grant et al. [10]	Umbrella review	“Each umbrella review focuses on a broad condition or problem for which there are two or more potential interventions and highlights reviews that address these potential interventions and their results.”	“…a response, and potential solution, to the perennial dilemma reviewers face regarding ‘lumping’ versus ‘splitting’, i.e., whether the needs of a particular field or area are best addressed by a broad review that covers multiple interventions at the cost of lost detail and specificity or by a succession of focused reviews that address specific comparisons at the risk of fragmenting the overall picture.”	“…compiling evidence from multiple reviews into one accessible and usable document.” “Graphical and tabular with narrative commentary”	“Identification of component reviews, but no search for primary studies”	None.

## Abbreviations

CADTH: Canadian Agency for Drugs and Technologies in Health
HTA: Health technology assessment
IHE: Institute of Health Economics
ITC: Indirect treatment comparison
MA: Meta-analysis
MeSH: Medical Subject Headings
NMA: Network meta-analysis
PICO: Population, intervention, comparator, and outcome
SR: Systematic review
